# Approaches in care for people with variations of sex characteristics—focus groups in the European context on the strengths and challenges of multidisciplinary teams

**DOI:** 10.1093/sexmed/qfae046

**Published:** 2024-08-13

**Authors:** Martin Gramc

**Affiliations:** Institute of Bioethics and History of Medicine, University of Zürich, Zürich 8006, Switzerland

**Keywords:** variations of sex characteristics, multidisciplinary care, peer support, psychosocial support

## Abstract

**Background:**

New guidelines in the *Consensus Statement on Management of Intersex Disorders* by the Lawson Wilkins Pediatric Endocrine Society/European Society for Paediatric Endocrinology Consensus Group 2006 introduced multidisciplinary teams (MDTs) to provide care that involves collaboration between healthcare professionals, parents, and children with variations of sex characteristics (VSC).

**Aim:**

The aim of this study was to examine a neglected but important field of collaboration among healthcare professionals and peer support groups who provide care for people with VSC.

**Outcomes:**

The study outcome was the information obtained regarding the actual composition and collaboration of the multidisciplinary teams caring for children with VSC, including their collaboration with parents, peer support groups, and other care providers.

**Methods:**

In this study we used an exploratory qualitative design based on mixed focus groups (in terms of professional background) and reflexive thematic analysis. Semi-structured focus group interview guides were used to obtain information about the participants’ viewpoints on the composition and challenges of, and collaboration between, peer support groups and members of multidisciplinary teams working to care for children with VSC and their parents. Seven focus groups were conducted with healthcare professionals and peer support groups in care teams in Central, Northern, and Western Europe. The data from the focus groups were examined using reflexive thematic analysis.

**Results:**

The participants in the focus groups provided information regarding the use of multidisciplinary and interdisciplinary child- and family-oriented approaches and the strengths and challenges of collaboration and peer support groups. The results showed that the teams used a predominantly multidisciplinary approach and suffered from a lack of psychosocial providers, poor collaboration with peer support groups, and poor implementation of shared decision-making to address the clinical uncertainty of parents and people with VSC.

**Clinical Implications:**

Clinicians should provide more psychosocial support and improve collaboration with peer support groups and nonmedical professionals.

**Strengths and Limitations:**

This study is one of the first qualitative studies to provide information on the collaboration of multidisciplinary teams working to provide care for children with VSC and collaborate with their parents in the European context. However, due to language barriers, the dropout rate of participants, and the under-representation of peer support groups in the sample there was a lack of information on collaboration among healthcare professionals and peer support groups.

**Conclusions:**

The collaboration between MDTs and parents does not involve adequate psychosocial and peer support or shared decision-making to address the uncertainty experienced by children and families when faced with information about variations of sex characteristics.

## Introduction

The *Consensus Statement on Management of Intersex Disorders* by the Lawson Wilkins Pediatric Endocrine Society/European Society for Paediatric Endocrinology Consensus Group^1^ published in 2006 introduced important recommendations for the care of people with variations of sex characteristics (VSC), with an emphasis on open and ongoing communication with families and the need for more holistic and multidisciplinary care. The statement paved the way for a more patient-oriented care approach for people with VSC, following a decade of critique by researchers, some healthcare professionals, and the individuals involved in the intersex movement.^2^^,^^3^ On the one hand, the consensus update from 2016 suggested caution regarding early, medically unnecessary surgical interventions.^4^ On the other hand, the 2016 update reinforced the idea of collaboration among the patient care team members and the provision of patient-centered care to enable patients and their parents to make fully informed decisions^4^ by drawing on respect for the participatory rights of children.^5^ Despite over 3 decades of proposed changes in practice and claims of improved care for people with VSC, the data obtained do not suggest that this shift has happened.^6^^,^^7^ In the years after the reported consensus statement, the critique continued as some intersex studies scholars and healthcare professionals began highlighting issues surrounding surgery and new terminology.^8-10^

According to the consensus statement, healthcare professionals are expected to communicate and collaborate with children and their parents in a shared decision-making process while educating other healthcare staff.^1^ The two main roles of multidisciplinary teams (MDTs) are to guide parents to accept the variations in their child while easing parental fears.^6^ Such teams can in fact be multidisciplinary, interdisciplinary, or transdisciplinary.^4^ According to the multidisciplinary approach, the services are separately planned among and provided by the members, and there is little or no communication among the team members.^4^^,^^11^ In multidisciplinary healthcare, professionals share a common goal: communication is clearly established through team leaders who are usually gatekeepers and whose role is to map out the required tasks and services. Skills and best practices come from different disciplines to redefine problems and solutions.^4^^,^^11^ An interdisciplinary approach refers to interconnections among different disciplines and professionals within and outside scientific disciplines.^12-14^ In interdisciplinary teams, the leader coordinates the management and collaborative communication of the team and develops interconnected plans based on needs as members solve problems across disciplines.^4^^,^^11^

Members from various disciplines are brought together in transdisciplinary teams. The roles of team members are blurred, and they are familiarized with the approaches of colleagues from other disciplines in order to be able to perform their roles to a certain extent as they share the responsibility for research and outcomes in practice.^4^^,^^15^

The reviewed literature suggests that the new multidisciplinary approach has not been adopted by all teams,^16^ and when it has been applied, it remains medically oriented, while psychosocial support of the collaboration among the team members remains neglected.^17^^,^^18^ The collaboration between children with VSC, their parents, and healthcare professionals is to be understood within the framework of pediatric shared decision-making as a process that is facilitated in order to reach a decision on treatment.^19^^,^^20^ The collaboration process has been increasingly conceptualized as multilateral because it obliges healthcare professionals to include various stakeholders, as well as their values and abilities, as equals.^21^

Psychosocial support and medicalization in care for people with VSC are also important concepts that must be highlighted. Psychosocial support attends to the emotional, psychological, spiritual, and social aspects of patients and their families,^22^ addressing the feelings and concerns of the patient as well as their families and other close relationswith the aim of improving the patient’s emotional and psychological well-being.^23^ Medicalization is a process of +-changing a nonmedical phenomenon into a medical problem.^24^^,^^25^ It is not absolute and can be good or bad.^25^ Good medicalization requires medical means to address medical issues.^25^ Bad medicalization unnecessarily transforms a social problem into a medical one, thereby recognizing human beings primarily as objects and neglecting their subjectivity.^25^

Little is known about the collaboration between multidisciplinary teams and the parents of a child with VSC.^26^^,^^27^ The few existing studies suggest that healthcare professionals sometimes disclose information in a way that parents find difficult to understand, presenting only a few options in decision-making and giving little time to process new information. Additionally, there is almost no adequate provision of psychosocial support for parents to cope with their emotions and to communicate with a child with VSC.^28-30^ Uncertainty primarily refers to scientific uncertainty because it addresses the uncertainties regarding diagnosis, causal explanations, and treatment recommendations.^31^ The uncertainty surrounding VSC in terms of how parents are included in the decision-making process remains unaddressed, as well as how VSC is still presented as a medical emergency, ie, an issue that has to be medically treated as soon as possible while neglecting support for parents that would address their worries and needs.^32^ Furthermore, there are only a few reported studies on collaboration among healthcare professionals.^33^^,^^34^ It remains unclear which care provider types are included in the team, how healthcare professionals collaborate with each other, and how these professionals cooperate with people with VSC and their families and peer support groups.

The present study was performed to examines the still-neglected field of collaboration among the stakeholders in caring for people with VSC as proposed by the original and updated *Consensus Statement on Management of Intersex Disorders*. This study examines the viewpoints of healthcare professionals and peer support group members dedicated to children with VSC and their families by highlighting the composition, organization, strengths, and challenges of multidisciplinary teams. I explored the collaboration dynamics among healthcare providers, peer support groups, and parents. The research questions guiding this study were the following: How have the recommendations from the consensus statement regarding collaboration in multidisciplinary teams been implemented, and what is the role of peer support groups in such process?

## Materials and methods

### Design

An exploratory qualitative design was used for this study, based on mixed focus groups (in terms of professional background) and reflexive thematic analysis.^35^ Due to this flexible interpretative approach, reflexive thematic analysis was used to capture participant viewpoints on experiences and practices of healthcare professionals and peer support groups in caring for children with VSC and their parents. These viewpoints were then critically examined to determine meaningful patterns and divergences.^35^ The research questions were addressed within constructionist, experiential, inductive, critical, and latent frameworks. The theoretical framework that informed the study design and guided the reflexive thematic analysis encompassed concepts of collaborative care in pediatric shared decision-making/collaboration,^20^ multidisciplinarity/professionalism,^12^^,^^21^ and children’s rights in pediatric medicine.^36^ Because reflexive thematic analysis is a heterogeneous approach that can take many forms, it is well suited to address the diversity of the concepts used in this study (multi/interdisciplinarity, medicalization, uncertainty, psychosocial support). A semi-structured guide to focus group interviews was used to obtain information about the participants’ viewpoints on the composition, collaboration, and challenges of peer support groups and the members of multidisciplinary teams involved in the care of children with VSC and their parents. The author of the present study is part of a broader research project called INIA—Intersex New Interdisciplinary Approaches—in which many intersex people are involved, but the author is not a person with VSC. Participation in the project enabled the author to become familiar with the issues involved in caring for people with VSC and the relevant stakeholders. The INIA project participants aim to develop knowledge that will inform policymaking and practices that support the wellbeing and social and economic contributions of people with VSC. The research project is informed by an applied research approach and the themes of the study could thus prompt actionable outcomes in the care of children with VSC.

### Selection of participants

The inclusion criteria for participation in the study were the following: participants had to be healthcare professionals working in a European multidisciplinary team providing care for children with VSC and their parents and/or members of peer support groups involved in any capacity in collaboration with a multidisciplinary team. Specifically, healthcare professionals were required to be medical specialists (primarily endocrinologists and urologists) or psychosocial support providers (psychologists and psychiatrists) who specialized in care for children with VSC.

A purposive selection procedure was used to select participants in the field of medical care for people with VSC on a European level.

Every focus group included a team coordinator. Four team coordinators were recruited during an in-person meeting at the 9th International Symposium on Disorders/Differences [DSDs] of Sex Development, where the author of this study participated in a workshop facilitated by their first supervisor. Three team coordinators and the team members, as well members of peer support groups, were recruited separately at the 9th DSD Symposium, ie, through snowballing. Two members of one peer support group were recruited by the team coordinators, who had been recruited at the 9th DSD Symposium, and one member of a different peer support group was separately recruited by the author. The team coordinators then took the initiative to contact other team members. Efforts were made to recruit participants from different countries.

### Data collection and data analysis

Seven focus groups in six different European countries (Belgium, Germany, Slovenia, Sweden, Switzerland, and the United Kingdom) were conducted from May 2022 to February 2023 with members of multidisciplinary teams and members of peer support groups. All focus groups were conducted by the author, who is a PhD candidate with a background in sociology and gender studies and 3 years of experience in qualitative research. The author developed the interview guide based on a scoping review^17^ and an additional literature search together with the second supervisor. The participants were sent information sheets and informed consent sheets 1 week before the scheduled time slot for the focus group and were asked to send them filled out and signed to the author. Focus group sessions lasted from 45 to 75 minutes, and all of them were conducted online on Zoom and were audio recorded. The average number of participants in the focus groups was 3 to 4. The focus groups were recorded with an iPhone SE using the Voice Memos app.

At the beginning of each session, the author explained to the participants the goals of the focus groups and presented the rules of the discussion. Then, the participants introduced themselves and explained their reasons for participating. This introduction was followed by asking the focus groups questions and receiving responses. In the event of unclear answers, the author asked for clarification. At the end of each focus group, the author gave the participants a chance to ask or state questions or concerns that arose during the discussion but had not been addressed. Finally, the author thanked the participants again for their collaboration and gave a final reminder to send their signed informed consent sheets.

The focus groups were conducted after a pilot study that included a focus group with one of the multidisciplinary teams contacted by the first supervisor and a member of a peer support group. After the pilot study, the author reduced the number of follow-up questions in the semi-structured interview guide (see Supplement 1) and reduced the number of main questions from 8 to 7 to make the focus groups less time-consuming with fewer redundant answers. Three focus groups were conducted in German and four in English, as the author is proficient in both languages. All the participants were either native or fluent speakers of the language used.

The focus group discussions were then transcribed and pseudonymized. The transcripts were not sent to the participants due to time constraints. The transcripts were coded with the program MAXQDA 2020 using the coding tree (Table 1). The author familiarized themself with the data by independently reading and taking notes on their first thoughts regarding the most common themes. Then, the second supervisor brainstormed with the author on adding codes. Afterward, the author and the second supervisor were joined by the first supervisor to compare their views on coding and to reflect on the assessment, which resulted in creating the first draft of the coding tree. The coding tree was adapted after the second reading of the transcripts and making notes on the previously determined provisionary themes and subthemes, while staying open to changes. The coding tree was designed inductively by multiple extensive readings of the transcripts and using the author’s notes made while conducting the focus groups. The codes were then qualitatively analyzed using reflexive thematic analysis as the author became familiar with the data in order to generate the themes.^5^

**Table 1:** Coding tree.

**Coding Tree**
Team
Team > Team composition
Team > Team composition > First contact
Team > Team composition > Tasks in the team
Team > Team composition > Discipline involved
Team > Team composition > Variation/individual dependent
Team > Team composition > Strengths
Team > Team composition > Weaknesses
Team > Physical location of the team and specialists
Team > Disciplinary boundaries
Team > Disciplinary boundaries > Positionality
Team > Disciplinary boundaries > Importance of the expertise
Team > Disciplinary boundaries > Transfer of knowledge
Team > Disciplinary boundaries > Awareness of knowledge differences/gap
Team > Responsibility for the treatment decisions
Team > Jurisdiction of the team
Team > Care approach
Team > Care approach > Medicalized
Team > Care approach > Demedicalized/pathologized
Team > Care approach > Multidisciplinary
Team > Care approach > Transdisciplinary
Team > Care approach > Interdisciplinary
Team > Care approach > Child-Oriented
Team > Care approach > Family oriented
Shared decision-making
Shared decision-making > Collaboration
Shared decision-making > Collaboration > Process
Shared decision-making > Collaboration > Trust
Shared decision-making > Collaboration > Based on the relationship with parents
Shared decision-making > Collaboration > Includes exchange of opinions among (all) the stakeholders
Shared decision-making > Collaboration > Hierarchical/horizontal
Shared decision-making > Collaboration > Hierarchical/horizontal > Between MDT and family
Shared decision-making > Collaboration > Hierarchical/horizontal > Among MDT members
Shared decision-making > Treatment decisions
Shared decision-making > Treatment decisions > Based on evidence
Shared decision-making > Treatment decisions > Among team members
Shared decision-making > Treatment decisions > Between team member(s) and patients/parents
Shared decision-making > Treatment decisions > Involves meetings and talking among MDTs and family
Shared decision-making > Treatment decisions > Deferral of a treatment
Shared decision-making > Treatment decisions > Who makes the decision
Shared decision-making > Conflicts
Psychosocial support
Psychosocial support > Care provider
Psychosocial support > Education of parents/patients
Psychosocial support > MDTs see psychosocial support important for the parents
Psychosocial support > Resources dependent
Psychosocial support > Addressing uncertainty
Psychosocial support > Supportive/addresses worries and needs
Psychosocial support > Referral
Psychosocial support > Mention of word intersex
Psychosocial support > Proactive attitude
Psychosocial support > Parental emotions
Peer support
Peer support > Collaboration with the team
Peer support > Reluctance
Peer support > Lack of peer support groups
Peer support > Integrated in the team?
Peer support > Role
Peer support > Role > Sensibility
Peer support > Role > Empowerment
Peer support > Role > Advisory for parents
Peer support > Role > Reduces loneliness/fears
Peer support > Role > Parents and/or people with VSC feel understood

Abbreviation: MDT, multidisciplinary team; VSC, variations of sex characteristics.

First, the author focused on the commonalities in participants’ reflections. Then, the author consulted with the research team on generating the themes. In the next stage, the author looked for similarities, differences, and commonalities in the transcribed material in order to create themes regarding medicalization and the role of knowledge. The author then re-read the material to ensure that the developed themes still had meaning and to see if the data could be understood in a way that engendered a third theme. This additional round of reading produced the third theme—uncertainty. The themes were then illustrated by a selection of quotations, which were slightly revised to improve readability and are included below. The quotes from focus groups conducted in German were manually translated by the author into English.

Ethics approval from the CEBES (Checkliste für den Ethik-Begutachtungsprozess von nichtbewilligungspflichtigen empirischen Studien) Review Board was obtained in February 2022, the ethics committee of the Institute of Biomedical Ethics and History of Medicine, University of Zurich. Approval code: 2022-01-CEBES-Review_INIA_GRAMC, date of approval: March 1, 2022. In January 2024 CEBES has been replaced by Ethics Committee of Univeristy of Zürich process: https://www.med.uzh.ch/en/Ethikkommission.html.

Not all of the people who initially expressed an interest in participating in the study were included in the final selection, as 5 individuals could not attend, 2 ultimately dropped out, 2 did not provide informed consent, and 1 decided to leave the focus groups at the beginning of the session.

## Results

The results are structured according to the following themes: medicalization, the role of knowledge, and uncertainty.

### Theme 1: medicalization

“Medicalization” refers to the composition of the teams, the division of labor, the importance of establishing diagnosis, and approaches to care.

#### Composition of the team

The participants reported that the teams providing care for people with VSC were predominantly composed of healthcare professionals, primarily pediatric endocrinologists, urologists/surgeons, gynecologists, and psychologists, the latter of whom are the main source of psychosocial support for parents (Table 2). In some cases, the composition of the teams depends on the variation at issue. A few teams included other healthcare professionals, such as neonatologists, geneticists, and pediatric nurses. This was summarized by one of the participants in the following way:

**Table 2:** Representation of the participants.

**Participants**	**Participant role**	**No. of participants**
Total participants		27
Healthcare professionals		23
	Endocrinologist	8
	Urologist/surgeon	5
	Psychologist	7
	Neonatologist	1
	Psychiatrist	1
	Gynecologist	1
	Ethicist	1
Group member	Peer support	3

**Table 3:** Themes and subthemes on the care for people with VSC in the European context.

**Themes**	**Medicalization**	**Role of knowledge (professionals)**	**Uncertainty**
Subthemes	Composition of teams	Provision of information	Treatment decisions
	Strengths and challenges	Importance of expertise	Parental emotions
	Primacy of diagnosis	The role of peer support groups	Positionality and responsibility for the treatment
	Division of labor		Psychosocial support
	Care approaches		

Abbreviation: VSC, variations of sex characteristics.

In our opinion, our team is represented by pediatric endocrinology together with psychology. So, we work very closely together as a team. But we also have a social worker in our psychosocial center and then we work closely with other specialist departments, ie, pediatric and adolescent gynecology, pediatric urology, and pediatric surgery, when discussing a case. (Endocrinologist, focus group [FG] 3)

This quote suggests that a high degree of representation of healthcare professionals in a team leads to good collaboration among and involvement by healthcare professionals, as they share the same professional background.

#### Division of labor

Endocrinologists and, to a lesser extent, psychologists are typically the first to contact parents. After the parents meet with the endocrinologist, who is usually the team leader, they are referred to a psychologist if the psychologist is not automatically invited to the first meeting with the parents. This way of communicating with parents is established in the protocol in most of the teams. There are larger and smaller team meetings in terms of the different medical specialties involved and the medical needs of people with VSC and their parents. In most teams, the division of labor is organized such that each specialist does what their specialty requires them to do, then meets with other professionals in team meetings to discuss the variation, diagnosis, and treatment pathways.

The quality of collaboration among healthcare professionals in the teams was further supported by a well-organized structure, long-established relationships, and openness to different opinions from other healthcare professionals. The overrepresentation of healthcare professionals and lack of psychosocial support providers and peer support groups indicate a discrepancy between the team focus and parental needs. One member of the peer support groups pointed out the following:

Apparently, there are three issues in this multidisciplinary team and these are medical issues, hormonal issues, and psychological issues. But when I hear, when I read what kind concerns the parents have… These are things like school, like giving information. How to find [relevant] children’s books. (A member of a peer support group, FG 7)

This quote by a member of a peer support group shows that multidisciplinary teams focus on medical and psychological issues, but the parental worries primarily revolve around the psychosocial aspects of the child’s development.

#### The primacy of diagnosis

According to the description of most participants, the diagnosis focuses on the communication of information about a given variation and treatment options for parents and children as soon as they are old enough to understand what it means to have VSC. Communication about the diagnosis is usually the starting point of collaboration among the team members, children with VSC, and their parents. The communication process is described as a process that involves many meetings and exchanges of opinions about the diagnosis and possible (non)treatment in the team, including disagreements and meetings with people with VSC and their family.

The diagnosis is conveyed to parents after the team has assembled and discussed the diagnosis of the person with VSC, as described by one of the focus group members:

So, we have a nice booklet for the parents when the child is born, so that they can somehow give us time, so that we can gain some time to make the best decision without pressure. And then we do examinations and try to get as close to the diagnosis as possible or get a diagnosis even genetically and as soon as possible to get an opinion as to what the best solution for the child would be. And then of course we also explain everything to the parents and then we decide together what the best option would be. They can ask questions, they can present opinions and dilemmas, and we discuss all of this. And as I said, a psychologist is also included very soon in the process. (An endocrinologist, FG 1)

As this quote illustrates, the way that a diagnosis is conveyed to parents echoes the composition of the team, as it is healthcare professionals—not psychosocial support providers or peer support groupswho provide the parents with information about the diagnosis. At this stage of collaboration among healthcare professionals and parents, it is the perspective of healthcare professionals as to the aims of care that takes precedence over parental concerns and wishes.

#### Care approaches

Most participants considered the care approach to be multidisciplinary since the team leader sets the tasks, which are then separately carried out by team members according to their medical specialties. This involves little communication or sharing of practices outside team meetings between the team members. However, the transfer of knowledge among team members was highlighted as important for the enrichment of the expertise and care of other team members, overall patient care, and the emerging interdisciplinary approach mentioned (sometimes interchangeably referred to as a multidisciplinary approach) by the participants in two focus groups. In the two teams, the members work with each other by integrating knowledge and practice from other disciplines in their work and to some degree also provide care that is not a part of their specialty.

In other words, it’s about interdisciplinarity, meaning a common language made up from different sub-specialties that formulate a common answer for the person seeking advice. (A psychologist, FG 2)

The interdisciplinary approach is highlighted by sharing vocabulary and knowledge from different subspecialties. However, the underrepresentation of providers of psychosocial support and peer support groups in the process of sharing knowledge and practices calls into question the larger shift to interdisciplinary care.

### Theme 2: the role of knowledge

The role of knowledge (professionals) primarily entailed the provision of information, the importance of expertise, and the knowledge and care provided by peer support groups.

#### The provision of information

According to the description of most participants, the provision of information is seen as important for the collaboration process. It focuses on diagnosis and treatment options for parents and children as soon as they are old enough to understand what it means to have VSC. Communication with parents happens after the team has assembled and discussed the diagnosis of the person with VSC. The information is provided by each healthcare professional, if parents wish, in the collaboration process with the team. However, information regarding psychosocial needs and support only featured prominently in a minority of the teams interviewed. As one of the participants pointed out:

Early information and early psychosocial support are extremely important for the growing child, even more than ever, as we want them to be part of the decision-making team as early as possible. And how can you be part of this team if you are not well informed? So, what we really aim at is trying to explain the different conditions already from 4 years onwards. That’s more or less the milestone. Well, depending, of course, a little bit individually, but we try to talk about the bodies and development already at that age and each time again at every visit. (An endocrinologist, FG 5)

The early provision of information related to psychosocial development is considered increasingly important, but the emphasis on information has to be taken cautiously as the over-representation of healthcare professionals in the study obscures the perspective of peer support groups and parents who are largely missing in the sample.

#### The importance of expertise

Most healthcare professionals in the study highlighted the importance of expertise in the provision of care. Expertise was referred to as important primarily regarding treatment decisions, supporting parents in their understanding of variations of sex characteristics, and the professional identity of team members.

The study participants mentioned two interconnected ways in which expertise plays a role in the team. First, expertise is seen as important in the transfer of knowledge, as the senior colleagues educate junior members from the same medical specialty. This process was closely related to team members frequently stating how they reflect on their own role in the team and the general role of the team. Second, expertise is seen as important for educating members of the team who do not belong to the same specialty, such that they can gain the understanding necessary to make decisions about future treatments. The latter point was highlighted by one of the participants as follows:

These are people with complex needs, and nobody has expertise in all aspects of the care of these people. So, you need people with different expertise to come together and contribute so that you can provide a holistic care approach to the patient. (An endocrinologist, FG 6)

There is the aspiration to provide comprehensive care to address the complexity of the needs of people with VSC, as the complexity of variations require different professionals. Yet, the question remains how team members should successfully address the complexity of the given needs considering the lack of nonmedical professionals in the team.

#### The role of peer support groups

According to the participants, psychologists usually refer parents to peer support groups, which in some teams are also an important part of educational training for parents. The participants also mentioned that peer support groups provide the kind of support and information that psychologists in the team cannot. For instance, peer support groups allow for the sharing of personal experiences and everyday worries, thus easing parental loneliness and fears about the uncertainty of the child’s development in the future. As one of the participants highlighted:

If you have questions, there is a support group which can help you and maybe together you can find some kind of solution or, for instance, a specialist to go to who is able to help you, if you don’t already have a good specialist or specialist in general. So, yes, a support group can do things for patients. (Member of a peer support group, FG 5)

This quote indicates that members of peer support groups are aware of the advantages of their input in the collaboration process with healthcare professionals and parents, but their invisibility might be an obstacle to reaching out to teams and parents.

On one hand, all participants commented that peer support groups are a scarce resource. On the other, they stressed that parents are often reluctant to meet them. Following the experiences expressed in the focus groups, peer support groups are not integrated in most of the MDTs, even though some healthcare professionals expressed a desire to include them in the team as the patient perspective is missing in the decision-making process. As one healthcare professional stated:

And I would also be very happy if we could move towards a situation where support groups were somehow part of our hospital team as well. We miss that perspective in our care model, and it would be really enriching to have that aspect as well. (An endocrinologist, FG 5)

The above quote indicates that there is willingness among some healthcare professionals to collaborate with peer support groups, which signals openness to demedicalized and cross-disciplinary collaboration.

However, some healthcare professionals in the focus groups expressed reluctance to include peer support groups, explaining that their inclusion could lead to a possible disruption of teamwork and decision-making. The participants clarified that support groups thus mainly collaborate with the MDTs in educational training programs for people with VSC and their parents. However, some of the participants expressed reluctance to include peer support groups in shared decision-making:

But for support groups to be a part of a formal shared decision-making process, let’s say within the MDT [meetings], which we’re holding, that’s a little bit dodgy because you’re going outside the governance procedure of our healthcare provider, which is our NHS Health Board. It’s very difficult then to bring somebody external who doesn’t have a contract with the Health Board to actually then come in and provide advice and be involved in that advice. (An endocrinologist, FG 6)

The resistance among some healthcare professionals to include peer support groups in the collaboration process signals that healthcare professionals consider peer support to be a service that is not part of the care provided by the teams. Healthcare professionals seem to consider the medicalized approach to be central.

### Theme 3: uncertainty

Uncertainty was pointed out regarding decisions about treatment options, responsibility for treatment options, parental emotions, and the role of psychosocial support in addressing uncertainty.

#### Treatment options

When discussing the treatment decision, the participants highlighted the primacy of biomedical assessment, on the one hand, and on the other, the difficulties of basing decisions on evidence—which, in the field of VSC, is lacking or contradictory. Some healthcare professionals thus admitted that they often do not know what the best treatment option might be, as described by this healthcare professional:

I was thinking about the fact that we tried to work in an evidence-based manner, but in this field it’s very difficult because the evidence is sometimes only emerging and sometimes changing and sometimes lacking and there is a vast field to keep track of. So, I could never keep track of all the different aspects, and I think no one could absorb all the new information on somebody else’s field. So, it’s quite important that the patients get the most accurate information from each of the team members. (A psychologist, FG 4)

This quote illustrates the issues regarding making sense of the data among healthcare professionals, because the complexity of the data drives the uncertainty about the suitable way to provide information and treatment options. A minority of healthcare professionals in the study stated that the lack of evidence and related uncertainty lead them to defer surgical interventions in early childhood, admitting that they often do not know what the best option is.

#### Parental emotions

Healthcare professionals in the study stated that they perceive parental emotions to reflect much uncertainty, shock, grief, and fear. The majority of healthcare professionals in the study expressed that parents struggle with understanding VSC, as in this statement:

The child is mostly healthy and merry, and finds out about it a lot later, that they are a bit different, but the parents see that in our gender binary way of thinking, which leads to anxiety, stigma, discrimination, and similar things. (A psychologist, FG 2)

The perception of healthcare professionals must be taken cautiously since they might misunderstand the emotional states in parents and disregard parental perspectives and experiences by deeming parents to be overwhelmed by emotions. In other teams, similar parental emotions are facilitated by providing psychosocial support that aims to explain information and handle parental emotions. It is noteworthy that while healthcare professionals mentioned parental emotions, they did not mention theirs.

#### Psychosocial support

Psychosocial support was mostly referred to as an explanation of information about VSC and emotional support for parents: “To meet parents where they are” (A psychologist, FG 4). It was usually mentioned when participants were talking about the collaboration process. The participants highlighted that psychosocial support is not always provided from the first contact with parents in all teams but is becoming increasingly central. According to their experience, psychosocial support is primarily provided by psychologists from MDTs and to a lesser degree by other healthcare professionals (endocrinologists and pediatric nurses). Psychosocial support is neither equally nor substantially integrated in the care provided by teams; psychologists were mentioned as a scarce resource, and the teams predominantly consist of medical professionals, some of whom misunderstand the role of psychologists. As one respondents pointed out:

Psychology is a scarce resource and actually psychologists spend a lot of time thinking and talking, not a lot of time seeing patients generally, unless you have a really good one. (A urologist, FG 6)

This quote reveals that some healthcare professionals do not understand the importance of psychosocial support, as it is considered to be simply thinking and talking.” Healthcare professionals perceive psychosocial support to be very important for parents, but they believe that parents do not understand the value of psychosocial support, as the following quote illustrates:

No, it really is an absolute pillar next to children’s endocrine care. And if the families don’t see it that way—I don’t think they do at first, they often come to us in pediatric endocrinology because they think okay, now let’s tell them medically and that’s how it goes on. (An endocrinologist, FG 3)

The participants highlighted their view that psychosocial support plays a crucial role in addressing the uncertainty that parents face when a child with VSC is born. The support is aimed at parental acceptance of the variation from the beginning, as reported by the members of the MDTs. The interviewed healthcare professionals stated that parents experience a range of emotions, such as fear, grief, stress, uneasiness, and shock related to uncertainty, because they regard parents as incapable of understanding VSC. Psychosocial support is aimed at addressing and reducing worries about the body image, sexual life, and relationships of people with VSC. The participants stressed that the issue of uncertainty is also related to the positionality of MDTs and their awareness of knowledge gaps, because the lack of knowledge or conflicting evidence makes the MDT members more cautious in their suggestions about treatment. The interviewed members of MDTs proactively advocate for psychosocial support. Healthcare professionals perceive that parents experience uncertainty in a distressful way. The distress healthcare professionals perceive in parents seems to lead healthcare professionals to steer the decision-making process in a paternalistic way, as one of the healthcare professionals stated:

If the parents are not with us, it is either because they disagree or because they don’t understand, and they just are blocked. So, we tried to help them with the emotional issues and understanding. We try to facilitate their learning and understanding of the situation. And that means that we are, well, in a way you could say that there we try to make them see things the way we do, but I don’t think we see it from one position. (A urologist, FG 4)

It is important to note that healthcare professionals refer to parental emotions but exclude their emotional experience when they refer to uncertainty in the decision-making process. However, parents are not necessarily distressed by the uncertainty, as they cope well with the variation in their child but may have worries about the child’s future that healthcare professionals are not able to address.

## Discussion

The purpose of this study was to assess collaboration between healthcare professionals and peer support groups in the care of children with VSC. The study has revealed a lack of ongoing and all-encompassing collaboration between healthcare professionals, children with VSC, their parents, and peer support groups in the care of children with VSC and their families in the European context. Most medical teams use a multidisciplinary approach, ie, team members apply knowledge and skills of their specific discipline, before or after meeting with other healthcare professionals from other disciplines to discuss the variation, the diagnosis, and how to proceed with the treatment. The results of our study are supported by Kyriakou et al.,^33^ who stated that even though a multidisciplinary approach had been introduced, only 40% of all the teams in their study used such an approach. This finding also points to a collaborative approach that first focuses on biomedical assessment and medical tools, whereas psychosocial support and a focus on the subjective experiences of children with VSC come in second. The focus on a biomedical assessment and the primacy of medical tools also indicates that the healthcare professionals in the teams primarily focus on the clinical aspects while sidelining care aimed at empowering children and their parents. This finding is in line with previous research by Liao and Roen,^18^ who pointed out the focus on biomedical practice and the sidelining of psychosocial support. Moreover, previous studies also indicated a demand for psychosocial support from parents that remains unmet.^29^^,^^37^

The excessive focus on clinical aspects blurs the line between good and bad medicalization,^25^ as the necessary medical examinations aimed at establishing the diagnosis and assessing the need for intervention come first. More time and resources are devoted to diagnosis and treatment than to care focused on the subjective experiences and empowerment of children and their parents. The lack of interdisciplinarity and psychosocial support additionally contribute to insufficiently demedicalized care, as scientific knowledge is not used for the empowerment of users of medical services.^25^^,^^38^ The inadequate implementation of interdisciplinary care could also be ascribed to the fact that the healthcare professionals in the study interchangeably used the terms “interdisciplinary care” and “multidisciplinary care,” signaling a lack of conceptual difference. The reason why the terms multidisciplinary and interdisciplinary are used interchangeably is also most likely due to the fact that healthcare professionals might understand the word “inter” as meaning “between.” Interdisciplinary thus simply refers to collaboration between different healthcare professionals.

Psychosocial tools and support are lacking not only because there are not enough resources (ie, psychologists), but also because there is reluctance to include peer support in the collaboration process. The perception of healthcare professionals provides only a partial picture of the reasons why parents seem to be reluctant to accept psychosocial support. Parents might be interested in seeking support outside a hospital, or they may be discouraged by the technical and medical information that healthcare professionals offer as the first step in care. Moreover, the communication among the team members is collaborative, but the lack of inclusion of non-medical professionals, such as peer support groups, which are already scarce tools for children with VSC and their parents, and the lack of collaboration with the team signals an inadequately implemented interdisciplinary approach. The insufficient collaboration between team members and peer support groups is consistent with previous findings on clinical practice. Namely, more than two-thirds of the teams involved in care for children with VSC and their parents do not have peer support available, and only one in three healthcare professionals is not aware of peer support groups.^33^^,^^34^

Another drawback in communication among team members and parents that arose in the focus groups was the lack of attention to parental emotions, such as distress and uncertainty, when told about VSC. It is important to note that healthcare professionals refer to parental emotions but exclude their emotional experience when they refer to uncertainty in the decision-making process. However, parents are not necessarily distressed by the uncertainty as they may cope well with a given variation but may have worries about the child’s future which healthcare professionals are not able to address. This suggests that collaboration among MDTs, people with VSC, and their parents is impaired by a lack of psychosocial support, which contributes to parental decisional conflict and a misunderstanding of conflicts and benefits, as indicated in research on parental decision-making.^39^ The fact that it was mainly healthcare professionals, but not the parents or their peer support representatives, who stated that parents were well integrated into the collaboration process, suggests that collaboration between parents and healthcare professionals is based on the assumptions of the latter. This finding is also consistent with a recent study on surgical decision-making for people with VSC that showed the need for healthcare professionals to be aware of parental needs, understanding, and worries, but highlighted the disagreement as to what degree parents should be involved in the decision-making process.^40^ Furthermore, the findings on the lack of addressing parental needs and worries in the shared decision-making process in this study are consistent with the recent research on clinical practices in care for children with VSC in North America, which stressed that only half of the teams discussed family values and understanding.^41^

The uncertainty and related emotions in parents also provide an opportunity for healthcare professionals to influence parental decision-making. Research on contraception and antenatal screening use shows that healthcare professionals have a great deal of latitude in constructing and presenting uncertainty.^42^^,^^43^ Furthermore, the analysis of decision-making practices in transgender medicine shows that conveying uncertainty where there is little or conflicting evidence leads healthcare professionals to performatively cooperate in decision-making while asserting medical authority, which closely resembles a paternalistic model of decision-making.^44^ The findings on the management of uncertainty and evidence in transgender medicine and antenatal testing provide useful analogies for decision-making in the care of children with VSC, as healthcare professionals in care for children with VSC continue to struggle with uncertainty^21^ in the current data and struggle to distinguish facts from assumptions.

Additionally, parents who have children with VSC are often left alone to make sense of biomedical assessments for which, unlike medical professionals, they do not possess the knowledge and expertise. The findings on the emotional states of parents with whom VSC are discussed are consistent with those of previous studies that revealed struggling with uncertainty, grief, shame, isolation, and uneasiness in the emotional experiences of parents when presented with the fact that their child has VSC.^45-47^

However, in some of the teams that participated in the focus groups in this study, psychosocial support was provided from the start and played a central role in the provision of information for addressing parental needs and emotions regarding uncertainty and collaboration with the team. This observation is consistent with previous findings on parents with children with complex medical conditions who overcame and were more empowered to navigate the unfamiliarity and uncertainty in the collaboration process when provided with continuous support and extensive education about the child’s condition.^48^ In a minority of teams in the sample, a psychologist was mentioned as the team coordinator, someone who accompanies families through the entire process. As one of the participants in the study pointed out, there is an emerging collaborative interdisciplinary approach to care for children with VSC and their parents in teams where psychosocial support takes a central role, including input from peer support groups. Nevertheless, in contrast to certain regions of Australia, European teams have not invested in establishing healthcare models that center around peer support and are community owned.^49^ European teams can learn from Australian colleagues how to coordinate care between healthcare professionals and the community of people with VSC, centering the human rights perspective, implementing new bioethical frameworks, prioritizing psychosocial support, aiding caregivers, combating stigma, and facilitating individuals in comprehending and articulating their treatment preferences and values.

## Limitations

One limitation of this study was participant recruitment, which led to not all of the focus groups being conducted in the mother tongues of the participants. A further limitation was that due to selection bias, peer support groups are absent from the sample, even though many were asked to participate. As parents were not selected for the sample, the information about their experiences in the analysis must be taken cautiously. The sample and analysis therefore privileged authoritative voices in the care for children with VSC and their parents, as the recruitment process was aimed at members who were healthcare professionals. Due to such past tensions, peer support groups were likely deterred from participating. The dropout of some participants also significantly influenced the final sample and the material used for the analysis. One of the research strategies that might improve the participation of peer support groups is to include one member of a peer support group among the MDT participants.

## Conclusions

The decision-making process for children with VSC and their parents lacks adequate psychosocial support and insufficiently addresses uncertainty surrounding information about VSC for parents. The care approach remains multidisciplinary, even though there is an emergent interdisciplinary approach. Teams are mainly composed of medical professionals who work separately, but more collaborative and task-sharing approaches are promisingly underway in a minority of teams. Collaboration among multidisciplinary teams and peer support groups is inadequate, lacking the inclusion of peer support groups and adequate psychosocial support that would help parents overcome the uncertainty regarding information about their child’s VSC. Future studies could explore in depth the relations between teams and peer support groups and how to include peer support groups in the shared decision-making process.

## Supplementary Material

Supplement_1_Interview_guide_version_7_with_statements_copy_qfae046

## Data Availability

The data that support the findings of this study are available from the corresponding author upon reasonable request.
