# Supplementary material for: Approaches in care for people with variations of sex characteristics—focus groups in the European context on the strengths and challenges of multidisciplinary teams
Source: Sex Med. 2024 Aug 13;12(4):qfae046. doi: 10.1093/sexmed/qfae046 (PMC11792176; doi:10.1093/sexmed/qfae046)
Supplement: Supplement_1_Interview_guide_version_7_with_statements_copy_qfae046 [file supplement_1_interview_guide_version_7_with_statements_copy_qfae046.docx]

**Interview guide**

Key phrases and words:

- decision-making

- treatment

- task

- parental role

- barriers and benefits to accessing support

- issues faced by parents

- best interest of the child

- mediation

- information/understanding

- collaboration

*Thank you all for volunteering to participate in this focus group. We are grateful for the time you are allocating to this research project. You have been invited to participate in this study because we value your opinions and experiences, which we deem essential for the successful conduct of this project.*

• Explain the research objectives of the study

• Explain the ground rules of the focus group (confidentiality – one participant speaks at the time/no overlap – agreement on terminology: VSC)

• Explain who is included into criteria of MDT, psychosocial support

• Role of moderators

• Ask if there are any questions on the process

*First, I kindly ask you to introduce yourselves, and briefly explain your motivation to participate in this discussion. This will help in breaking the ice and getting to know better each other a bit more.*

*--*

*Thank you for introducing yourself! Our first question would be*:

1) If I say, multidisciplinary team, what do you think of?

*A multidisciplinary team utilises the skills and experience of individuals from different disciplines, with each discipline approaching the patient from its own perspective.*

*Interdisciplinary teamwork synthesises distinct contributions from different approaches into a single consultation model without confusing professional boundaries (Liao and Simmonds).*

2) In your opinion, what is the primary role or function of multidisciplinary teams working in the care of children with variations of sex characteristics and their families? Who should ideally be part of the MDT?

*The majority of psychologists (11) and some general practitioners (6) did not have*

*a strong opinion about surgery. Instead, they focused on parental decision-making*

*and the importance of shared informed consent with families and parents. While still*

*disregarding children’s opinions and bodily autonomy, the health professionals’ role*

*was described as central, but subordinate to a primary responsibility to parents (Prandelli and Testoni).*

3) Based on your personal experiences, how do you collaborate with the members of MDT in the decision-making process?

Possible follow-up questions:

- The decisions made by health care providers are based on what data or evidence?

- What works well? What would you change?

- What kind of challenges have you experienced when collaborating, within and outside of the groups present at the table? Especially in the conflicting situations?

- Who makes the final call in conflicting situation?

- What kind of role psychosocial support plays in the decision-making process?

*There are many surprising, challenging interactions between parents and medical staff, parents and their children’s bodies, parents and their children, parents and their social environment, and so on (Meoded Danon and Krämer).*

4) In your opinion, what kind of support families of children with VSC receive?

If there is anything that could be improved, what would it be?

Sub-questions/prompts:

- Professional support

- Peer support/ Advocacy groups

- When the support should be introduced?

*The physical and psychological health needs for individuals with a DSD are closely interrelated. Medical management decisions in DSD are often based on promoting psychological adaptation and well-being rather than a physical health need per se. (Brain et. al.)*

5) What are your thoughts on the following statement?

*“The parents and the medical team must assign gender. It is paradoxical situation because the major decision is taken without consulting the patient themself and without knowing what the wishes of the patient are going to be.”*

6) How would you describe the role of support groups in relation to decision-making process? What challenges and opportunities might there be?

Sub-questions/prompts:

- What are the barriers and how might these be overcome?

- What are the goals of parents for reaching out to such groups?

- How parents contact such groups?

*Feelings of isolation and/or stigmatization are reduced by meeting similar others (Davies & Hall, 2005).*

7) What are your experiences and thoughts on transitional care, transfer of medical care from childhood to adulthood?

*How­ever, although models of best practice have been established in paediatric care services for those with a DSD, ensuring continuity of care into adulthood remains problematic (Naomi S. Crouch and Sarah M. Creighton)*

8) Is there anything else that you would like to add that was not addressed before?
